# The psychological mechanism of basic public service provision affecting population urbanization in China: a moderated mediation model based on migrants’ settlement intention

**DOI:** 10.3389/fpsyg.2026.1887684

**Published:** 2026-08-13

**Authors:** Zerui Ni, Yan Jin

**Affiliations:** 1 School of Public Administration, Huaiyin Normal University, Huai'an, China; 2 School of Social Development and Public Policy, Fudan University, Shanghai, China; 3 School of Marxism, Shanghai Lixin University of Accounting and Finance, Shanghai, China

**Keywords:** basic public service provision, migrants’ settlement intention, moderated mediation model, population urbanization, psychological mechanism

## Abstract

**Objective:**

The influence of social welfare and psychological factors is becoming increasingly prominent in China’s people-oriented new-type urbanization. However, how macro-level public service provision translates into urbanization outcomes through psychological mechanisms remains poorly understood.

**Materials and methods:**

This study introduces migrants’ settlement intention (MSI), a psychological construct capturing expectations, social belonging, and future-oriented decisions, as a mediator between basic public service provision (BPSP) and population urbanization (PU) in China. Using panel data from 291 Chinese prefectural cities and the China Migrants Dynamic Survey (2014–2018), this study employs a moderated mediation model to examine how BPSP affects PU through MSI.

**Results:**

Findings indicate that MSI partially and positively mediates the BPSP-PU relationship after overcoming the endogeneity issues, with significant and positive paths from BPSP to MSI (*β* = 0.368, *p* < 0.01) and from MSI to PU (*β* = 0.005, *p* < 0.05). Besides, housing price negatively moderates this mediation (*β* = −0.149, *p* < 0.05; *β* = −0.035, *p* < 0.05), while hukou restrictions negatively moderate the BPSP-MSI link (*β* = −0.296, *p* < 0.01) but positively moderate the MSI-PU link (*β* = 0.242, *p* < 0.01). These mediating and moderating effects remain reliable and consistent across a series of robustness checks. Heterogeneity analyses further reveal that: First, livelihood-related services not only amplify the mediating effect of MSI between BPSP and PU, but also enhance the negative moderating effect of hukou restrictions. Second, MSI shows a suppression effect on PU in Central China and has a significant positive mediating effect in Northeast China. Moreover, housing price and hukou restrictions moderate the mediation most strongly in Eastern and Western China, respectively. Third, MSI mediates the BPSP-PU link more positively and significantly in peripheral cities. Housing price moderates more negatively in central cities, and hukou restrictions moderate more strongly in peripheral cities.

**Conclusion:**

By identifying MSI as a forward-looking psychological conduit, this study advances a psychological account of urbanization and underscores the value of integrating urban psychology with public policy to foster sustainable, human-centered urban development.

## 1 Introduction

The world has experienced rapid population urbanization (PU), with over 80 per cent of the world population now residing in urban areas, and this share is projected to keep rising (United Nations, 2025). This rapid urbanization is largely driven by migration and natural urban growth (Zimmer et al., 2026), particularly in developing countries such as China. Since the reform and opening-up, China’s urbanization rate surged from 17.92% in 1978 to 67% in 2024, with permanent urban residents exceeding 943.5 million (NBS, 2025). Although this rapid population growth in cities promotes socioeconomic development, it has also triggered challenges including PU lagging behind land urbanization and industrialization (Zhou et al., 2025; Luo et al., 2020), urban–rural and regional disparities (Liu et al., 2022), and semi-urbanization (Wang et al., 2025). These problems pose severe threats to sustainable urban development and human wellbeing (Han J. et al., 2021; Yang et al., 2024). As China’s urbanization enters a high-quality development stage, PU now plays an important role in promoting high-quality economic development and socialist modernization. Therefore, it is crucial to pay attention to the improvement of PU, which has been regarded as a fundamental strategy for achieving people-centered new-type urbanization in China.

Basic public services are pivotal in raising living standards, enhancing social equity, and achieving common prosperity (Cheng et al., 2025). Recently, basic public service provision (BPSP) in China has steadily improved. However, several problems sill persist including unbalanced development (Li G. et al., 2024), inefficient and polarized provision (Ji et al., 2024), and insufficient service (Guo et al., 2026; Mai et al., 2025). These issues intensify rural–urban dualism, increase governance risks, and hinder urbanization, especially in underdeveloped regions. To further promote the high-quality development of PU, China promulgated the National New-type Urbanization Plan (2014–2020) in 2014 and the 14th Five-Year Plan for New-type Urbanization in 2022, emphasizing a shift from land-centered to people-centered urbanization and highlighting the focus of urbanization from economic growth to the equalization of basic public services (Gu et al., 2025). New-type urbanization (NTU) requires improving public service to meet citizens’ growing needs for a better life and foster urban sustainability (Yuan et al., 2025). Consequently, the role of BPSP has historically transformed from hukou-related thresholds to retention levers for population integration, with differentiated and compensatory BPSP driving spatial population redistribution.

Some studies have examined the impact of BPSP on migration or urbanization. Tiebout (1956) first incorporated basic public services into migration analysis through the “voting with feet” theory, arguing that free migrants tend to choose regions where public services and taxation best suits their preferences. Many studies have confirmed that imbalanced and hierarchical differences in BPSP shape migration decisions and PU processes (Liu et al., 2025; Yang et al., 2026). In terms of influencing mechanisms, migrants’ settlement intention (MSI), as an antecedent variable in their decision-making of migration behavior, reflects both BPSP effectiveness and PU development potential. The BPSP is positively correlated with the living quality, development expectations and the sense of social belonging and social integration of the migrants (Li et al., 2024b), which in turn profoundly shapes their psychological inclination to reside long-term in urban areas. Therefore, MSI ultimately constitutes a critical psychological bridge and behavioral precursor linking BPSP to the behaviors of migrants, such as permanent residence, home purchase, and urban hukou acquisition (Zhu, 2007).

However, most existing studies have examined the effects of income inequality, climate change, and other factors on population urbanization (PU) from economic or ecological perspectives (Han B. et al., 2021; Chen and Goutte, 2026), with much less attention paid to the role of basic public services. Even among the few studies that touch upon the relationship between public services and population mobility, their analysis largely remains at the macro-level effect estimation and fails to delve into the underlying transmission mechanisms or heterogeneity. More critically, scant research has approached this issue from migrants’ psychological perspective to systematically investigate how basic public service provision (BPSP) shapes population urbanization (PU) through its influence on individual settlement intentions. This psychological mediation pathway remains virtually uncharted in the urban psychology literature. In addition, the existing empirical evidence is constrained by notable data and methodological limitations. Most studies rely on provincial-level panel data or cross-sectional surveys, which are unable to capture the spatial dynamics at the prefecture level and largely overlook the potential endogeneity between BPSP and PU. These limitations not only undermine credible causal inference but also leave the micro-level psychological mechanisms underlying urbanization processes long obscured and poorly understood, with relevant research remaining largely unexplored.

To address the above research gaps, this study makes three primary contributions. First, from the interdisciplinary perspective of urban psychology and public policy, it innovatively establishes migrants’ settlement intention (MSI) as a core psychological mediating variable, systematically revealing the underlying pathway through which basic public service provision (BPSP) translates into population urbanization (PU) outcomes by shaping individuals’ cognitive appraisals and emotional identification. This mechanism remains largely unexplored in the urban psychology literature, thereby filling an important theoretical gap. Second, it constructs a moderated mediation model to examine the moderating roles of housing price and hukou restrictions at different stages of the mediation process, deepening our understanding of how capitalization of public services and institutional constraints jointly shape the micro-level mechanisms of urbanization. Third, it conducts heterogeneity analyses across three dimensions, namely public service types, geographic regions, and city administrative levels, revealing the differential patterns of the psychological mediation effect under varying macro-level contexts and providing empirical evidence for the formulation of targeted policies on equalization of basic public services and hukou reform. By elucidating the psychological transmission pathway from public services to urbanization, this study not only advances a psychological account of urbanization but also offers theoretical support and policy implications for achieving people-centered new-type urbanization. Therefore, using data from 291 Chinese prefecture-level cities and the China Migrants Dynamic Survey (2014–2018), this study constructs a theoretical framework and employed a moderated mediation model to examine whether MSI mediates the BPSP-PU relationship and whether the mediating effects are moderated by housing price and hukou restrictions. The heterogeneity of the impact mechanisms across different BPS types, regions, and city administrative levels were further examined. The endogeneity bias was addressed and several robustness tests were also performed to confirm the credibility of the main findings.

## 2 Theoretical framework and research hypotheses

Traditional migration theory highlights economic motivations as primary drivers, where individuals migrate based on cost–benefit calculations for better employment and income (Lewis, 1954; Todaro, 1969). Tiebout (1956) firstly put forward the theory of “vote with their feet,” suggesting migrants choose regions with public services best suiting their preferences. The provision of abundant public services is posited to play a crucial role in enhancing migrants’ wellbeing and fostering their long-term settlement intentions (Liu et al., 2025; Wen et al., 2022). However, to fully understand this process, it is imperative to move beyond a purely economic cost–benefit calculus and adopt a psychological lens. Such a lens explicates how macro-level public service inputs are translated into individual-level migratory decisions. Drawing upon environmental psychology and social identity theory, this study identifies migrants’ settlement intention (MSI) as the core psychological mechanism. MSI is a forward-looking cognitive and affective construct that encapsulates future expectations, place attachment, and social belonging (Zhou et al., 2024). Unlike transient emotional states, MSI represents a stabilized psychological orientation towards a specific locale (Zou et al., 2024). This makes it a critical and immediate precursor to actual urbanization behaviors, such as family relocation, home purchasing, and hukou acquisition (Lu et al., 2024).

From a psychological perspective, BPSP operates through two interrelated pathways to shape MSI. The first is the cognitive-evaluative pathway. Public services in education, healthcare, and social security serve as salient environmental affordances that reduce existential uncertainties and future risks (Stark and Taylor, 1991). According to expectation-value theory and the cognitive appraisal framework, migrants form positive appraisals of their own life prospects and upward mobility in that city when they perceive that a city provides superior and accessible public services. This perceived “life feasibility” directly elevates their goal-oriented intention to settle (Liu et al., 2024; Zhang et al., 2026). The second is the affective-identitarian pathway. Drawing on place attachment theory and the social identity approach, BPSP, particularly in cultural, recreational, and community domains, facilitates social interactions, fosters a sense of community integration, and strengthens migrants’ emotional bonds with the host city (Zhang et al., 2026; Wood et al., 2025). As migrants’ daily routines become embedded within the locally provided service ecosystem, they develop a psychological sense of “being at home.” This sense is a potent driver of long-term residential commitment (Lyu, 2024). Thus, BPSP enhances MSI not merely by lowering material costs, but also by satisfying fundamental psychological needs for security, competence, and relatedness. These needs are essential for transforming a temporary sojourner into a permanent urban citizen.

Furthermore, the transition from heightened MSI to actual population urbanization (PU) is also contingent upon the realization of collective psychological capital. As MSI becomes widespread among the migrant population, this aggregated psychological inclination generates a self-reinforcing virtuous cycle (Li et al., 2024a). A critical mass of migrants with strong settlement intentions will pool human capital, diversify the local labor market, and stimulate consumption. The “scale effect” brought by this process will also invigorate the city’s economic dynamism, ultimately enhancing urban productivity and PU development (Chen et al., 2023; Firmino Costa da Silva et al., 2017). Moreover, from a policy feedback perspective, municipalities with a high concentration of intending settlers face increased public pressure to further equalize service provision and improve governance. This pressure not only accelerates the conversion of intentions into actual permanent residence but also creates a more inclusive institutional climate that sustains urbanization (Liu et al., 2024). That is, higher settlement intentions encourage migrants to shift from short-term employment to long-term stable residence, which can expand the labor market and activate domestic demand, thereby supporting urban development (Adunts et al., 2026). This top-down (policy to psychology) and bottom-up (psychology to macro-outcome) interplay underscores the pivotal bridging function of MSI. In essence, MSI serves as the critical psychological conduit, a filtering mechanism that translates the objective availability of public services into the subjective commitment necessary for population urbanization. Without this psychological mediation, the effects of BPSP may remain latent or dissipate due to perceptual filters, institutional frictions, or a lack of emotional investment. Accordingly, we proposed that:

*Hypothesis 1*: MSI positively mediates the relationship between BPSP and PU.

Although areas with higher BPSP can attract more population inflows and promote PU, the associated rise in housing prices may counteract this effect by increasing living costs and weakening settlement intentions. The capitalization of public services into land prices will significantly increase migrants’ housing expenditure (Feng and Lu, 2013), including rent and down payments in urban areas, thereby reducing their disposable income and living quality, and weakening their willingness to settle. Moreover, the housing wealth effect exacerbates resource allocation inequality and limits migrants’ equal access to services, further diminishing their social integration, migration motivation and settlement intentions (Li et al., 2025; Murphy et al., 2006). Excessive housing price increases may also misallocate resources toward the real estate sector, crowding out investment in other productive industries, decreasing the potential for urban socioeconomic growth, and ultimately hindering the realization of MSI and the sustainable advancement of PU. Therefore, we proposed that:

*Hypothesis 2*: Housing price negatively moderates the relationship between BPSP and MSI, and the relationship between MSI and PU.

Besides, hukou restrictions may negatively affect the mediation process of MSI. BPSP in China is related to the registered residence system, preventing rural–urban migrants from accessing public services equally (Yuan et al., 2015). Most migrants only experience occupational and geographic mobility, but lack the same access to public services and social welfare as local hukou-holding residents (Wu and Yang, 2025). This discrimination increases migrants’ implicit survival costs and decreases their development opportunities (Huang and Ke, 2024), consequently weakening MSI and PU. Furthermore, the hukou system emphasizes social stratification and constantly generates new social differences, leading to a new “dual structure” determined by hukou status within cities. This may weaken migrants’ sense of belonging and identity, thereby reducing migration stability and integrity. The instability and uncertainty of migration caused by hukou controls also undermines urban economies of scale, resulting in significant efficiency losses that may weaken the positive mediating effect of MSI. These institutional barriers may trigger feelings of relative deprivation and social exclusion, which are key psychological mechanisms that erode settlement intentions and hinder the translation of intentions into actual urbanization.

On the contrary, hukou restrictions may also positively influence MSI and PU by avoiding rapid declines in per capita resources and enhancing living stability. Appropriate hukou restrictions can regulate population inflow, mitigate labor market competition and prevent excessive dilution of social welfare caused by short-term migrant influxes (Hu et al., 2025). Meanwhile, regions with stricter hukou restrictions generally provide better services, thus increasing their attractiveness (Sheng et al., 2026). Additionally, the “reverse selection” effect of hukou restrictions helps screen immigrants by attracting and retaining high-skilled migrants while discouraging the entry of low-skilled migrants (Fan et al., 2009). This will optimize population structure and spatial distribution within cities, improve resource *allocation efficiency, and promote the development of high-tech industries, ultimately facilitating MSI* and PU. We therefore hypothesized that:

*Hypothesis 3*: The mediating effect of MSI is moderated by hukou restrictions.

**Figure 1:**
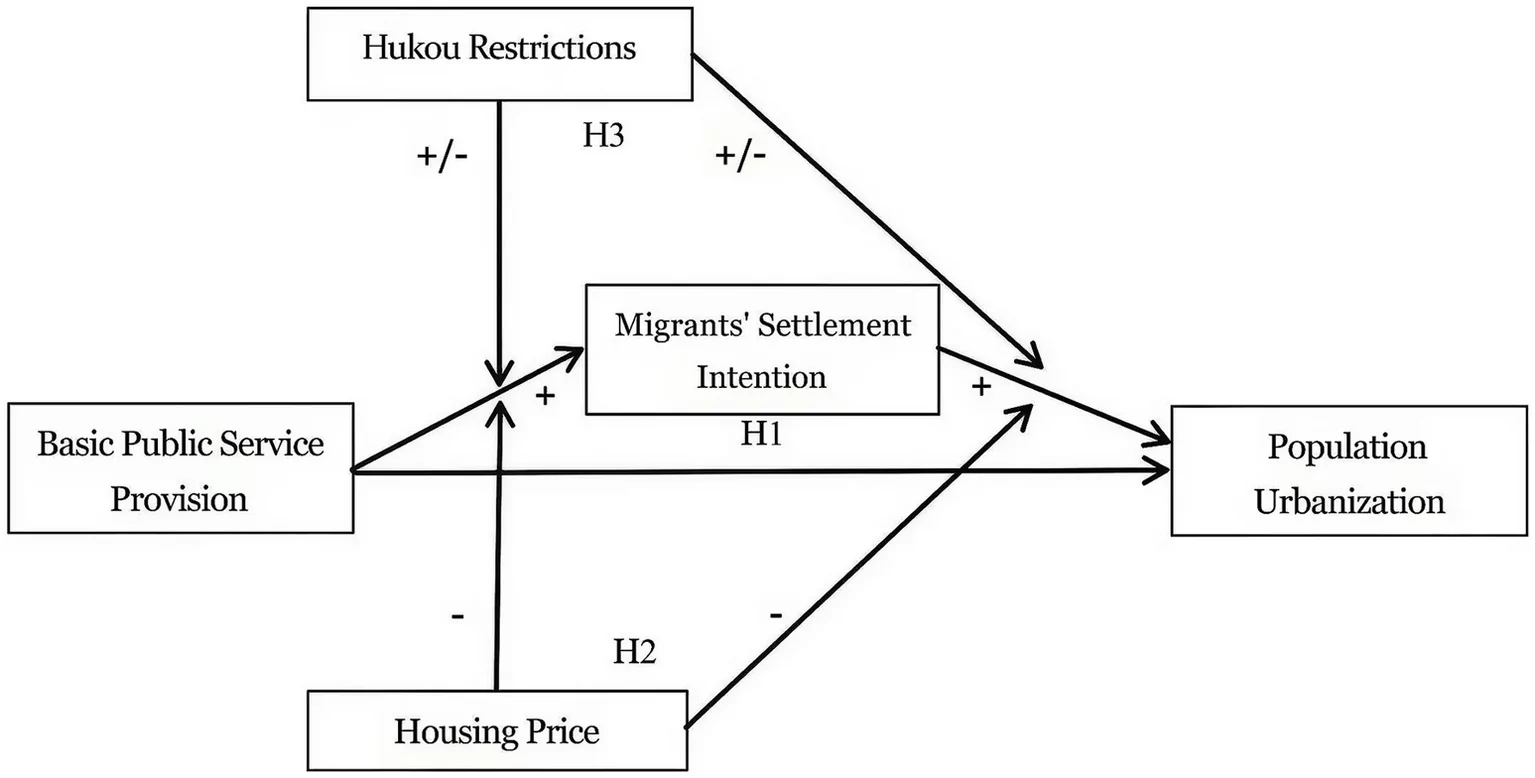
Theoretical framework.

Figure 1 summarizes the relationships among these hypotheses.

## 3 Research methods

### 3.1 Econometric model

#### 3.1.1 Baseline model

Following Wen and Ye (2014), this study employs the stepwise method to test the mediating effect of MSI (see Equations 1–3):


$$PU_{\mathrm{it}}=\alpha_{0}+\alpha_{1}\mathrm{BPS}P_{\mathrm{it}}+\gamma_{1}\text{controls}+\delta_{i}+\mu_{t}+\varepsilon_{\mathrm{it}}\;\;$$ (1)

$$\mathrm{MS}I_{\mathrm{it}}=\chi_{0}+\chi_{1}\mathrm{BPS}P_{\mathrm{it}}+\gamma_{2}\text{controls}+\delta_{i}+\mu_{t}+\varepsilon_{\mathrm{it}}$$ (2)

$$\begin{array}{c} PU_{\mathrm{it}}=\lambda_{0}+\lambda_{1}\mathrm{BPS}P_{\mathrm{it}}+\lambda_{2}\mathrm{MS}I_{\mathrm{it}}+\gamma_{3}\text{controls}+\delta_{i}+\mu_{t}+\varepsilon_{\mathrm{it}} \end{array}$$ (3)

Where *i* and *t* represent city and year, respectively. *δ_i_* represents the city fixed effect, *μ_t_* represents the time fixed effect, and *ε_it_* denotes the random error term. Additionally, *α_1_* is the total effect of BPSP on PU, *χ_1_* is the effect of BPSP on MSI, *λ_1_* is the direct effect of BPSP on PU, *λ_2_* is the effect of MSI on PU. If *α_1_*, *χ_1_* and *λ_2_* are all statistically significant, it indicates that BPSP affects PU through MSI. Moreover, if *λ_1_* is significant, it suggests a partial mediating effect; otherwise, it represents a full mediating effect.

#### 3.1.2 Moderated mediation model

To examine the moderating effects of housing price and hukou restrictions on both the BPSP-MSI and MSI-PU relationships, this study constructs the moderated mediation model and strictly follows the analytical paradigm proposed by Wen and Ye (2014). The moderated mediation model has been widely applied in research fields such as psychology, economics, and public administration (Lu et al., 2024). Specifically, we added interaction terms between these two moderators and the corresponding independent variables (BPSP and MSI) into the models:


$$\begin{array}{c} \mathrm{MS}I_{\mathrm{it}}=\beta_{0}+\beta_{1}\mathrm{BPS}P_{\mathrm{it}}+\beta_{2}\text{pric}e_{\mathrm{it}}+\beta_{3}\text{huko}u_{\mathrm{it}}+\beta_{4}\mathrm{BPS}P_{\mathrm{it}}\times \text{pric}e_{\mathrm{it}}\\ +\beta_{5}\mathrm{BPS}P_{\mathrm{it}}\times \text{huko}u_{\mathrm{it}}+\gamma_{4}\text{controls}+\delta_{i}+\mu_{t}+\varepsilon_{\mathrm{it}} \end{array}$$ (4)

$$\begin{array}{c} PU_{\mathrm{it}}=\theta_{0}+\theta_{1}\mathrm{BPS}P_{\mathrm{it}}+\theta_{2}\mathrm{MS}I_{\mathrm{it}}+\theta_{3}\text{pric}e_{\mathrm{it}}+\theta_{4}\text{huko}u_{\mathrm{it}}\\ +\theta_{5}\mathrm{MS}I_{\mathrm{it}}\times \text{pric}e_{\mathrm{it}}+\theta_{6}\mathrm{MS}I_{\mathrm{it}}\times \text{huko}u_{\mathrm{it}}\\ +\theta_{7}\mathrm{BPS}P_{\mathrm{it}}\times \text{pric}e_{\mathrm{it}}+\theta_{8}\mathrm{BPS}P_{\mathrm{it}}\times \text{huko}u_{\mathrm{it}}\\ +\gamma_{5}\text{controls}+\delta_{i}+\mu_{t}+\varepsilon_{\mathrm{it}} \end{array}$$ (5)

Where *price* and *hukou* are the moderators. *β* and *θ* are the estimated parameters. Equations 4, 5 were used to test the moderating effect of housing price and hukou restrictions on the relationship between BPSP and MSI, and the relationship between MSI and PU.

#### 3.1.3 Models for endogeneity treatment

To address potential bidirectional causality between BPSP and PU, this study further employs two approaches to mitigate potential endogeneity issues and check the mediating effect of MSI on the relationship between BPSP and PU. Firstly, we employed the one-period lag of BPSP as its instrumental variable and estimated the regression coefficients using 2SLS method. Theoretically, the lagged value serves as a valid instrumental variable for its contemporaneous counterpart. On the one hand, the lagged value of a variable is correlated with the latter. On the other hand, the lagged value is not related to the current disturbance term, as it has already been determined and is therefore exogenous to current shocks. It shows that the one-period lag of BPSP can only affect MSI or PU through BPSP. Secondly, the System Generalized Method of Moments (SGMM) estimation was further applied to dynamic panel data models, where the one-period lag of MSI and PU were selected as instrumental variables.

### 3.2 Variables and measurements

#### 3.2.1 Dependent variable

Population urbanization (PU). Population urbanization denotes the dynamic transformation process of population concentration in urban areas (Han et al., 2026). According to the relevant literature (Han et al., 2026; Gu et al., 2025; Yang et al., 2024), this study selects the proportion of urban permanent resident population to total population as a proxy variable for population urbanization. It should be further noted that, although urbanization is a complex process encompassing multiple dimensions, including economic, social, and spatial transformations, this article mainly focuses on the demographic dimension of urbanization. We adopted this measurement primarily based on the following considerations. First, population urbanization itself is the core essence of urbanization, and the urbanization rate can, to a large extent, reflects the fundamental profile of the urbanization process. Second, the proportion of urban population to total population remains the internationally recognized core indicator for measuring urbanization (United Nations, 2025), and directly corresponds to the key policy objective of “increasing the share of urban permanent residents” in China’s new-type urbanization strategy, thus possessing strong policy relevance and international comparability. Third, under China’s statistical definition, migrants who have resided in a city for more than 6 months are counted as permanent residents, meaning that this indicator inherently captures the dynamic process of rural-to-urban migration and settlement, rather than merely reflecting a static population size. Therefore, this indicator has significant advantages in terms of theoretical consistency, policy relevance, and cross-city comparability, making it a reasonable and valid dependent variable for this study.

#### 3.2.2 Independent variable

Basic public service provision (BPSP). Basic public services refer to essential services aimed at meeting residents’ basic needs, especially in safeguarding fundamental rights to survival, health and self-development (Yuan et al., 2025). Referring to the relevant literature and the14th Five-Year Plan (2021–2025) for promoting equalization of basic public services (Guan and Ma, 2025; Li G. et al., 2024; Zhang et al., 2023), this study selects 19 representative quantitative indicators to construct a systematic comprehensive evaluation system for BPSP from the seven dimensions of public education, medical and health care, social security, public culture, public infrastructure, scientific services, and environmental protection (see Table 1). This indicator selection follows the principles of scientific rigor, systematic coherence, representativeness, and operability, while also accounting for data structure, quality, accessibility, and China’s social-economic realities. Specifically, this study gives priority to per capita, per land area, or ratio-based relative indicators. These measures effectively eliminate the interference of urban administrative hierarchy and population size differences on scale effects. They also directly reflect residents’ actual accessibility to public services and the depth of benefits they receive. Meanwhile, this indicator set covers resource input, facility allocation, and governance performance. This design aims to systematically portray the level of public service provision from multiple stages, including input, output, and effectiveness. It also facilitates horizontal comparisons across cities and vertical tracking of trends over time.

**Table 1:** Evaluation index system of basic public service provision.

Target	Dimensions	Indicators	Attribute	Weight
Basic public service provision	Public education	Pupil-teacher ratio of regular primary schools	+	0.0249
		Pupil-teacher ratio of junior secondary schools	+	0.0234
	Medical & health care	Number of beds in hospitals and health care institutions per 10,000 people	+	0.0342
		Number of health technicians per 10,000 people	+	0.0281
	Social security	Number of urban employees covered by basic pension insurance per 10,000 people	+	0.0696
		Number of urban employees covered by basic medical insurance per 10,000 people	+	0.0734
		Number of people covered by unemployment insurance per 10,000 people	+	0.0897
	Public culture	Number of books in public libraries per 10,000 people	+	0.1016
	Public infrastructure	Number of highway mileage per square kilometer (road network density)	+	0.0449
		Number of public buses per 10,000 people	+	0.0819
		Number of taxis per 10,000 people	+	0.0812
		Water supply penetration rate of cities	+	0.0037
		Gas penetration rate of cities	+	0.005
	Scientific services	Per capita expenditures on science and technology	+	0.2381
	Environmental protection	Greening coverage rate of the built-up areas	+	0.0054
		Park green space per capita	+	0.0604
		Wastewater treatment rate	+	0.0109
		Harmless treatment rate of household waste	+	0.0061
		Comprehensive utilization rate of industrial solid waste	+	0.0174

Additionally, to ensure comparability across scales and years, we adopted the entropy weight method to calculate the weights and values of BPSP, which is highly regarded for its objectivity, comprehensiveness and simplicity (Wang et al., 2023). The measurement of BPSP was further converted into a relative index for robustness test. The calculation are as follows (see Equations 1–3).


$$\begin{array}{c} {\text{BPSP}}_{\mathrm{ij}}^{\prime }=\mathrm{LN}(\frac{{\text{BPSP}}_{\mathrm{ij}}-\bar{{\text{BPSP}}_{j}}}{\bar{{\text{BPSP}}_{j}}}+2) \end{array}$$ (6)

Where ${\text{BPSP}}_{\mathrm{ij}}^{\prime }\;$ is the disparity index for city *i* in year *j*, with higher values indicating higher BPSP; values above LN2 signify above the national average. $\bar{{\text{BPSP}}_{j}}$ denotes the national average BPSP across all cities in year *j*.

#### 3.2.3 Mediating variable

Migrants’ settlement intention (MSI). Migration or settlement decision is a rational process, with settlement intentions reflecting migrants’ expectations of the city (Jin et al., 2025). Following Chen and Luo (2022), we measured MSI using the CMDS question: “Do you intend to stay here long-term (more than 5 years)?” Responses “yes” were coded as 1 (intended) and “no/undecided” as 0 (not intended). City-level MSI is the proportion of “yes” responses among migrants in each city.

#### 3.2.4 Moderating variable

Housing price. It is a key cost factor that migrants must consider when relocating. It was measured by the ratio of total commercial housing sales to sales area.

Hukou restriction. It was measured by the ratio of permanent resident population to registered residence population (Han and Li, 2019). The larger the ratio, the higher the degree of deviation between two groups, indicating stricter hukou control. Given that permanent residents reflect the tax base and registered residents represent the fiscal burden, this indicator can capture local governments’ willingness to use hukou restrictions to balance fiscal accounts and protect financial interests.

#### 3.2.5 Control variable

To mitigate omitted variable bias, this study includes a series of control variables that may affect PU. Referring to previous relevant studies, we controlled for economic development, industrial structure, innovation level and population density (Cai et al., 2025; Lu et al., 2019). The theoretical basis and detailed measurement for control variables are as follows: (1) Economic development. Existing literature shows that the level of economic development is a fundamental driving factor for urbanization (Chen et al., 2022; He et al., 2016). In this study, we therefore used per capita real GDP (economic scale), the average wage of employed workers (wage income), the urban registered unemployment rate (unemployment level), per capita retail sales of social consumer goods (marketization level) and the ratio of actual utilized foreign capital to GDP (degree of openness) to measure economic development. By controlling for these variables, this study can better mitigate the impact of economic factors. (2) Industrial structure. According to Meng et al. (2024), industrial structure is directly related to new-type urbanization (Meng et al., 2024). Therefore, this study includes the ratio of tertiary-to-secondary sector output to measure industrial structure and mitigates the interference of industrial structure on PU. (3) Innovation level. Technological innovation serves as an indispensable driver for achieving high-quality new urbanization, exerting a significantly positive influence (Cai et al., 2025). Therefore, this paper chooses to control innovation level and employs the number of patent applications granted per 10,000 people to measure it. (4) Population density. Building on the research of Sun et al. (2024), population density promotes agglomeration economy and is closely related to urban development. We therefore used the ratio of the permanent residents to administrative area as a proxy for population density, thereby controlling for the potential influence of population agglomeration on PU. Besides, this study also controls for urban fixed effects and time fixed effects.

### 3.3 Data and study area

This paper uses a sample of 291 prefecture-level cities in China from 2014 to 2018, because the MSI data from CMDS is only available up to 2018. The data are sourced from the China City Statistical Yearbook (2015–2019), China Statistical Yearbook (2015–2019), China Urban Construction Statistical Yearbook (2014–2018) and the statistical yearbooks of various provinces and cities (2015–2019). MSI is derived from the 2014–2018 China Migrants Dynamic Survey (CMDS), conducted by National Health and Family Planning Commission of China using a stratified, multi-stage PPS sampling method, with sample size proportional to population size. It is worth noting that the CMDS data on migrants’ settlement intention at the city level have not been publicly released beyond 2018, making this the most recent year available for our analysis. Missing data were addressed through interpolation method to ensure completeness and continuity.

## 4 Results

### 4.1 Mediating effect of migrants’ settlement intention

Table 2 presents the results of mediating effect of MSI on BPSP and PU. Models 1 and 2 report the baseline mediation results based on stepwise method proposed by Wen and Ye (2014). Model 1 shows that BPSP significantly and positively influences MSI at the 1% significance level (*β* = 0.469, *p* < 0.01). Model 2 shows that both BPSP and MSI have significant positive effects on PU (*β* = 0.453, *p* < 0.01; *β* = 0.021, *p* < 0.1). These results provide preliminary evidence that MSI partially and positively mediates the BPSP-PU relationship.

**Table 2:** Mediating effect analysis: results of stepwise method and endogeneity test.

Variables	Baseline		2SLS		System GMM	
	Model 1	Model 2	Model 3	Model 4	Model 5	Model 6
	MSI	ln(PU)	ln(MSI)	ln(PU)	ln(MSI)	ln(PU)
ln(BPSP)	0.469*** (9.14)	0.453*** (20.52)	0.478*** (8.51)	0.488*** (19.97)	0.368*** (4.93)	0.102*** (4.71)
ln(MSI)		0.021* (1.92)		0.016+ (1.45)		0.005** (2.01)
L.ln(MSI)					0.262*** (3.87)	
L.ln(PU)						0.766*** (23.25)
Controls	YES	YES	YES	YES	YES	YES
City fixed effect	YES	YES				
Year fixed effect	YES	YES				
R-squared	0.143	0.742				
Prob>F			0.000	0.000		
AR (1) test *p*-value					0.000	0.001
AR (2) test *p-*value					0.153	0.824
Hansen test *p*-value					0.664	0.793
Observations	1,455	1,455	1,455	1,455	1,455	1,455

Values in parentheses are *t*-statistics. ***p < 0.01, **p < 0.05, *p < 0.1, ^+^*p* < 0.15.

Model 3–6 reports the results of endogeneity tests. Using the one-period lag of BPSP as an instrumental variable, the 2SLS estimation results (Models 3–4) suggest that MSI remains a positive mediator. The statistical results also show that the F-statistic of first-stage is greater than 10, indicating no weak instrument problem. Additionally, overidentification is not a concern when using a single instrumental variable. It indicates that the positive mediating effect of MSI on the BPSP-PU relationship is reliable after addressing endogeneity concerns.

The results of SGMM approach in Models 5–6 show that BPSP still has a positive and significant impact on MSI (*β* = 0.368, *p* < 0.01), and MSI significantly and positively influences PU (*β* = 0.005, *p* < 0.05). The empirical results further show that AR (1) is less than 0.1 and AR (2) is greater than 0.1, indicating that the differenced error term exhibits first-order serial correlation but no second-order serial correlation, thus rejecting the hypothesis of serial correlation in the error term of the level equation. The Hansen test *p*-value is greater than 0.1, suggesting no overidentification problem and that the instrumental variables are valid. These findings indicate that MSI partially mediates the relationship between BPSP and PU, which confirmed Hypothesis 1.

### 4.2 Moderating effects of housing price and hukou restrictions

To explore the moderating effects of housing price and hukou restrictions, we constructed a moderated mediation model (Table 3). In Model 1, the interaction term between BPSP and price is significantly negative (*β* = −0.149, *p* < 0.05). In Model 2, the interaction between MSI and price is also significantly negative (*β* = −0.035, *p* < 0.05). These results indicate that housing price negatively moderates both the BPSP-MSI and MSI-PU relationships, supporting Hypothesis 2.

**Table 3:** Results of moderated mediation effect.

Variable	Housing price		Hukou restrictions	
	Model 1	Model 2	Model 3	Model 4
	ln(MSI)	ln(PU)	ln(MSI)	ln(PU)
ln(BPSP)	0.573*** (11.87)	0.520*** (57.70)	0.588*** (14.76)	0.335*** (15.92)
ln(MSI)		0.015 (1.00)		0.037** (3.08)
ln(BPSP) × ln(price)	−0.149** (−4.53)	−0.149*** (−15.01)		
ln(BPSP) × ln(hukou)			−0.296*** (−4.84)	−0.377*** (−27.56)
ln(MSI) × ln(pri0ce)		−0.035** (−2.93)		
ln(MSI) × ln(hukou)				0.242*** (7.21)
Controls	YES	YES	YES	YES
City fixed effect	YES	YES	YES	YES
Year fixed effect	YES	YES	YES	YES
R-squared	0.178	0.756	0.165	0.784

Values in parentheses are *t*-statistics and all moderating variables have been centered. ***p < 0.01, **p < 0.05, *p < 0.1, ^+^*p* < 0.15.

The interaction between BPSP and hukou in Model 3 is significantly negative (*β* = −0.296, *p* < 0.01), while the interaction between MSI and hukou in Model 4 is significantly positive (*β* = 0.242, *p* < 0.01). These results suggest that hukou restrictions negatively moderate the BPSP-MSI relationship but positively moderate the MSI-PU relationship. Therefore, Hypothesis 3 was verified.

### 4.3 Robustness test

#### 4.3.1 Adopting alternative basic public service provision

To ensure the accuracy of the findings, the explanatory variable was first replaced for robustness test. The level of BPSP mentioned above was calculated based on the entropy weight method, which is an absolute numerical value. Referring to the method of Li et al. (2015), the absolute value was further converted into a relative index to measure BPSP. As shown in Model 1–2 of Table 4, the mediating effect of MSI remains significantly positive (*β* = 0.527, *p* < 0.1; *β* = 0.009, *p* < 0.01). The results of Model 3–4 also present that the moderating effects of housing price are mainly significant and negative (*β* = −0.182, *p* < 0.05; *β* = −0.008, *p* > 0.15). Moreover, the moderating effects of hukou restrictions are still significant (*β* = −0.263, *p* < 0.05; *β* = 0.257, *p* < 0.01). These findings are largely consistent with the baseline results.

**Table 4:** Robustness test: results of adopting alternative basic public service supply.

**Variable**	Model 1	Model 2	Model 3	Model 4	Model 5	Model 6
	ln(MSI)	ln(PU)	ln(MSI)	ln(PU)	ln(MSI)	ln(PU)
ln(BPSP)	0.527* (1.89)	0.173*** (6.29)				
ln(MSI)		0.009*** (3.10)				
ln(BPSP) × ln(price)			−0.182** (−4.48)	−0.172*** (−12.52)		
ln(BPSP) × ln(hukou)					−0.263** (−3.37)	−0.394*** (−9.93)
ln(MSI) × ln(price)				−0.008 (−0.79)		
ln(MSI) × ln(hukou)						0.257*** (7.59)
Controls	YES	YES	YES	YES	YES	YES
Observations	1,455	1,455	1,455	1,455	1,455	1,455

Values in parentheses are *t*-statistics and all moderating variables have been centered. ***p < 0.01, **p < 0.05, *p < 0.1, ^+^*p* < 0.15.

#### 4.3.2 Adjusting the sample

The migrants who planned to live locally for more than 5 years were considered as having settlement intentions in the 2014–2017 CMDS. However, the 2018 CMDS included the migrants who have lived in the place of immigration for 5 years. To ensure accuracy, we excluded the 2018 CMDS data and reconducted the regression using the 2014–2017 samples. The results in Table 5 show that BPSP significantly promotes PU through MSI (*β* = 0.443, *p* < 0.01; *β* = 0.006, *p* < 0.05). Housing price still negatively moderates both the BPSP-MSI and MSI-PU relationships (*β* = −0.117, *p* < 0.05; *β* = −0.033, *p* < 0.1). Hukou restrictions negatively moderate the BPSP-MSI relationship but positively moderate the MSI-PU relationship (*β* = −0.255, *p* < 0.05; *β* = 0.222, *p* < 0.01). It indicates that the mediating and moderating effects remain consistent with the baseline findings.

**Table 5:** Robustness test: results of adjusting the sample.

Variable	Model 1	Model 2	Model 3	Model 4	Model 5	Model 6
	ln(MSI)	ln(PU)	ln(MSI)	ln(PU)	ln(MSI)	ln(PU)
ln(BPSP)	0.443*** (5.37)	0.132*** (6.89)				
ln(MSI)		0.006** (2.05)				
ln(BPSP) × ln(price)			−0.117** (−4.80)	−0.149*** (−11.87)		
ln(BPSP) × ln(hukou)					−0.255** (−4.18)	−0.377*** (−19.88)
ln(MSI) × ln(price)				−0.033* (−2.52)		
ln(MSI) × ln(hukou)						0.222*** (8.51)
Controls	YES	YES	YES	YES	YES	YES
Observations	1,164	1,164	1,164	1,164	1,164	1,164

Values in parentheses are *t*-statistics and all moderating variables have been centered. ***p < 0.01, **p < 0.05, *p < 0.1, ^+^*p* < 0.15.

#### 4.3.3 Handling outlier

To mitigate the influence of extreme values, we applied a 1% truncation to all continuous variables. The results in Table 6 show that the mediating effect of MSI remains significantly positive (*β* = 0.503, *p* < 0.01; *β* = 0.021, *p* < 0.1) and the moderating effects of housing price (*β* = −0.211, *p* < 0.05; *β* = −0.036, *p* < 0.05) and hukou restrictions (*β* = −0.356, *p* < 0.05; *β* = 0.312, *p* < 0.01) are also significant. These results remain consistent with the baseline regression in terms of coefficient direction and significance, confirming the robustness of our findings.

**Table 6:** Robustness test: results of outlier handling.

Variable	Model 1	Model 2	Model 3	Model 4	Model 5	Model 6
	ln(MSI)	ln(PU)	ln(MSI)	ln(PU)	ln(MSI)	ln(PU)
ln(BPSP)	0.503*** (9.17)	0.462*** (18.41)				
ln(MSI)		0.021* (1.66)				
ln(BPSP) × ln(price)			−0.211** (−3.00)	−0.197*** (−8.27)		
ln(BPSP) × ln(hukou)					−0.356** (−2.21)	−0.488*** (−7.64)
ln(MSI) × ln(price)				−0.036** (−2.95)		
ln(MSI) × ln(hukou)						0.312*** (4.70)
Controls	YES	YES	YES	YES	YES	YES
Observations	1,236	1,236	1,236	1,236	1,236	1,236

Values in parentheses are *t*-statistics and all moderating variables have been centered. ***p < 0.01, **p < 0.05, *p < 0.1, ^+^*p* < 0.15.

### 4.4 Heterogeneity analysis

#### 4.4.1 Heterogeneity of basic public service types

Different types of basic public services vary in content, form and accessibility, leading to heterogeneous effects on MSI and PU. This study further examines the mediating role of MSI and the moderating effects across basic public service types. Following Han and Li (2019), basic public services are classified into livelihood-related services (e.g., public education, medical and healthcare, social security, public culture) and infrastructure-related services (e.g., public infrastructure, scientific services, environmental protection). Models 1–2 and 5–6 in Table 7 show that the effects of BPSP on MSI and MSI on PU are both positive and significant in livelihood-related public services (*β* = 0.511, *p* < 0.05; *β* = 0.008, *p* < 0.05), indicating that the mediating effect of MSI is more pronounced.

**Table 7:** Heterogeneity of basic public service types.

Variable	Livelihood-related public services				Infrastructure-related public services			
	Model 1	Model 2	Model 3	Model 4	Model 5	Model 6	Model 7	Model 8
	ln(MSI)	ln(PU)	ln(MSI)	ln(PU)	ln(MSI)	ln(PU)	ln(MSI)	ln(PU)
ln(BPSP)	0.511** (2.07)	0.169*** (7.05)			−0.146 (−1.39)	−0.020 (−1.10)		
ln(MSI)		0.008** (2.26)				0.010*** (2.80)		
ln(BPSP) × ln(price)			−0.230* (−1.93)	−0.084*** (−5.58)			−0.271** (−2.11)	−0.066*** (−3.23)
ln(BPSP) × ln(hukou)			−1.569*** (−4.04)	−0.178** (−2.56)			0.178 (0.52)	−0.079 (−1.26)
ln(MSI) × ln(price)				0.014 (1.57)				0.012 (1.37)
ln(MSI) × ln(hukou)				−0.015 (−0.90)				−0.005 (−0.26)
Controls	YES	YES	YES	YES	YES	YES	YES	YES

Values in parentheses are *t*-statistics and all moderating variables have been centered. ***p < 0.01, **p < 0.05, *p < 0.1, ^+^*p* < 0.15.

Models 3–4 and 7–8 in Table 7 further show that housing price negatively and significantly moderates the relationship between BPSP and MSI across different public service types (*β* = −0.230, *p* < 0.1; *β* = −0.271, *p* < 0.05). Besides, hukou restrictions have a prominent negative moderating effect on BPSP-MSI relationship in livelihood-related public services (*β* = −1.569, *p* < 0.01), which is significantly greater than that of housing price (*β* = −0.230, *p* < 0.1). These results indicate that the mediating and moderating effects are heterogeneous by basic public services types.

#### 4.4.2 Heterogeneity of geographic region

Given regional variations in economic structure, cultural customs, natural environments and the scale of migrants, the mediating and moderating effects may differ. To explore geographic heterogeneity, we divided China into four regions: East, Central, West, and Northeast. As shown in Table 8, MSI has a suppression effect on PU in Central China (*β* = −0.046, *p* < 0.01) and plays a significant positive mediating role in the Northeast (*β* = 0.108, *p* < 0.05), but shows no significant mediating effect in the East and West (*β* = 0.003, *p* > 0.15; *β* = −0.008, *p* > 0.15).

**Table 8:** Heterogeneity of geographic region.

Variable	Eastern region				Central region			
	Model 1	Model 2	Model 3	Model 4	Model 5	Model6	Model7	Model 8
	ln(MSI)	ln(PU)	ln(MSI)	ln(PU)	ln(MSI)	ln(PU)	ln(MSI)	ln(PU)
ln(BPSP)	0.457*** (3.56)	0.207*** (5.87)			0.485*** (4.19)	0.394*** (10.41)		
ln(MSI)		0.003 (0.23)				−0.046*** (−2.85)		
ln(BPSP) × ln(price)			−0.572** (−2.48)	−0.060*** (−3.28)			0.021 (0.06)	−0.135*** (−5.19)
ln(BPSP) × ln(hukou)			−0.245 (−0.27)	−0.209*** (−2.91)			0.101 (0.10)	−0.196** (−2.56)
ln(MSI) × ln(price)				0.039*** (3.08)				−0.007 (−0.40)
ln(MSI) × ln(hukou)				−0.023 (−1.14)				−0.044 (−1.07)
Controls	YES	YES	YES	YES	YES	YES	YES	YES

Variable	Western region				Northeastern region			
	Model 9	Model 10	Model 11	Model 12	Model 13	Model 14	Model 15	Model 16
	ln(MSI)	ln(PU)	ln(MSI)	ln(PU)	ln(PU)	ln(MSI)	ln(MSI)	ln(PU)
ln(BPSP)	0.383*** (4.44)	0.387*** (10.77)			0.228+ (1.49)	0.690*** (8.27)		
ln(MSI)		−0.008 (−0.39)				0.108** (2.49)		
ln(BPSP) × ln(price)			0.407 (1.22)	−0.058 (−1.58)			0.049 (0.06)	−0.048 (−0.60)
ln(BPSP) × ln(hukou)			−2.412** (−2.42)	−0.357*** (−3.37)			1.376 (0.33)	−0.609 (−1.48)
ln(MSI) × ln(price)				0.017 (0.74)				0.082 (1.62)
ln(MSI) × ln(hukou)				−0.011 (−0.26)				−0.166 (−1.35)
Controls	YES	YES	YES	YES	YES	YES	YES	YES

Values in parentheses are *t*-statistics and all moderating variables have been centered. ***p < 0.01, **p < 0.05, *p < 0.1, ^+^*p* < 0.15.

Additionally, this study analyzes regional differences in moderated mediating effects. As shown in Table 8, the negative moderating effect of housing price on the mediation chain is most pronounced in the eastern region (*β* = −0.572, *p* < 0.05; *β* = 0.039, *p* < 0.01), while the moderating effect of hukou restrictions is most evident in the western region (*β* = −2.412, *p* < 0.05; *β* = −0.011, *p* > 0.15). These findings confirm that the moderated mediation effects of MSI are diverse by geographic region.

#### 4.4.3 Heterogeneity of city administrative level

Under the administrative hierarchy system in China, cites with larger scale and higher administrative levels generally possess more service resources (Yu and Zhou, 2023). Therefore, this study categorizes cities into central (municipalities, provincial capitals, and sub-provincial cities) and peripheral cities, and explores the heterogeneous mediating effect of MSI across different city administrative levels. As shown in models 1–2 and 5–6 of Table 9, BPSP significantly promotes MSI and the impact of MSI on PU is significantly positive in peripheral cities (*β* = 0.479, *p* < 0.01; *β* = 0.017, *p* < 0.15), but this mediating effect is not significant in central cities (*β* = 0.263, *p* < 0.01; *β* = −0.008, *p* > 0.15).

**Table 9:** Heterogeneity of city administrative level.

Variable	Central cities				Peripheral cities			
	Model 1	Model 2	Model 3	Model 4	Model5	Model 6	Model 7	Model 8
	ln(MSI)	ln(PU)	ln(MSI)	ln(PU)	ln(MSI)	ln(PU)	ln(MSI)	ln(PU)
ln(BPSP)	0.263*** (5.94)	0.362*** (8.83)			0.479*** (10.68)	0.468*** (51.47)		
ln(MSI)		−0.008 (−0.32)				0.017+ (1.91)		
ln(BPSP) × ln(price)			−0.378** (−2.36)	−0.078** (−2.43)			−0.339* (−1.95)	−0.069*** (−4.50)
ln(BPSP) × ln(hukou)			0.122 (0.19)	−0.355*** (−2.97)			−1.712*** (−2.74)	−0.266*** (−4.81)
ln(MSI) × ln(price)				0.008 (0.22)				0.014* (1.78)
ln(MSI) × ln(hukou)				−0.006 (−0.08)				−0.015 (−0.81)
Controls	YES	YES	YES	YES	YES	YES	YES	YES

Values in parentheses are *t*-statistics and all moderating variables have been centered. ***p < 0.01, **p < 0.05, *p < 0.1, ^+^*p* < 0.15.

This study further analyzes the moderated mediating effects across cities of different administrative levels. As shown in Models 3–4 and 7–8 of Table 9, housing price exerts a stronger negative moderating effect on the mediation of MSI in central cities (*β* = −0.378, *p* < 0.05), while hukou restrictions have a more pronounced negative moderating effect in peripheral cities (*β* = −1.712, *p* < 0.01). It shows that the mediating and moderating effects are heterogeneous by city administrative levels.

## 5 Discussion

This study finds that that MSI partially and positively mediates the BPSP-PU relationship. Housing price negatively moderates this mediation, while hukou restrictions negatively moderate the BPSP-MSI link but positively moderate the MSI-PU link. Moreover, these mediating and moderating effects vary across public service types, regions and city administrative levels. These results provide a psychological explanation for the relationship between BPSP and PU, and offer a theoretical basis and practical reference for advancing people-oriented urbanization and promoting BPSP. Findings also demonstrate that mediating and moderating effects are consistent and reliable after conducting robust test. The stability of the mediation pathway across various endogeneity corrections reinforces this psychological mechanism, indicating that the observed relationships are not merely econometric artifacts but reflect genuine psychological processes.

Consistent with “voting with feet” theory, BPSP positively affects PU, with MSI partially mediating this relationship. These findings substantiate the view that migrants’ settlement intention functions as a core psychological channel, which transforms macro-level public service improvements into macro-level urbanization outcomes through individual-level psychological changes. It reveals that the impacts of welfare and public services are not only generated through policy thresholds, but also through the subjective interpretations or psychological channels (Wu and Yang, 2025). It further indicates that citizenization process encompass not only the physical movement of people but also the integration at social, economic, and cultural aspects (Wang and Shen, 2026). The increase in MSI related to BPSP contributes to citizenization, which in turn positively contributes to PU. It should be further noted that, given the mobility constraints imposed by the hukou system, the Pareto efficiency expectations of the Tiebout model are manifestly inapplicable to China’s institutional realities. However, this article believes that the value of the “voting with feet” theory mainly lies in the fact that differentiated public goods supply affects population spatial distribution, and it fits well with the research design of this article. On the one hand, Tiebout model suggests that this research design exploring the influence mechanism between basic public services and population urbanization (the outcome of rural population migration) is feasible. On the other hand, this article uses the “voting with feet” theory as a fundamental theoretical framework and combines it with other psychology and economics theories to explain the causal chain of “BPSP-MSI-PU.” This design does not require migrants to have complete mobility options, but only requires that their mobility decisions have a systematic response to the differences in basic public services between cities. Even though China’s household registration system has restrictions on mobility, this condition can still be met, especially among populations with strong mobility intentions. In addition, this article further discusses the moderating role of hukou restrictions in the following content, which can help us understand the impact of hukou system on the migration process in China.

This study also suggests that housing price negatively moderates the BPSP-MSI-PU relationship, and hukou restrictions negatively moderate the BPSP-MSI link but positively moderate the MSI-PU link. It indicates that high housing price increases the living expenditure of immigrants, so the benefits brought by increased BPSP are partially offset, leading to a decrease in settlement intentions and PU outcomes. This highlights that rising housing costs amplify perceived financial strain and residential insecurity (Li et al., 2025), which directly counteract the psychological benefits of basic public services such as enhanced social integration and life satisfaction (Lyu, 2024; Zhang et al., 2026), thereby eroding their settlement intentions and subsequent realization. Besides, strict hukou control limits migrants’ equal access to public services and reduces their sense of belonging (Zhao et al., 2025; Gu and Zhao, 2025; Vortherms and Liu, 2022), ultimately weakening the positive impact of basic public services on migrants’ willingness to stay. Also, a well-designed hukou system can serve as a screening mechanism to attract high-quality talents and promote the realization of MSI and high-quality urbanization. However, overly strict hukou control may decrease talents flow and hinder economic development (Li et al., 2022). Thus, balancing hukou control and openness is crucial.

Heterogeneity analysis further reveals three patterns. First, the mediating effect of MSI and the negative moderating effect of hukou restrictions are stronger for livelihood-related public services. A possible explanation is that livelihood-related public services are directly related to the migrants’ life quality and sense of belonging (Wood et al., 2025), while infrastructure-related public services reflect macro-level urban development potential and indirectly influence individuals’ emotional bonds and settlement intentions. This may also stem from the fact that hukou restrictions directly hinder migrant populations from accessing the same livelihood-related public services as local residents. Livelihood-related public services are often exclusive to registered residents and generate greater marginal impact on migrants (Zhang et al., 2026; Vortherms and Liu, 2022), while infrastructure-related public services characterized by strong universality and substitutability, such as transportation and environmental protection, have relatively limited marginal impact on migrants. Therefore, the positive impact of BPSP related to livelihood is more significantly weakened by hukou restrictions. These findings reveal that the psychological pathways from basic public services to urbanization are heterogeneous by service types. Livelihood-related services engage more deeply with the emotional and identity-based dimensions of settlement intentions, making the mediating effect of MSI both stronger and more susceptible to institutional barriers.

Second, the mediating effects of MSI on the BPSP-PU relationship are heterogeneous by geographic region, especially on the MSI-PU path. BPSP consistently and positively influences MSI in different regions, while MSI diversely affects PU. Specifically, MSI shows a suppression effect on PU in Central China and has a significant positive mediating effect in the Northeast, but shows no significant mediating effect in the eastern and western regions. This may be explained by the fact that Central China emphasizes the quantity-over-quality urban development model, where the level of urban management services is relatively low, and social conflicts and uneven distribution of resources are evident. In this context, the increased MSI which was boosted by BPSP, has not been effectively transformed into a driving force for population urbanization. Instead, high MSI may exacerbates the competition and instability of urban life, which in turn reduces PU. On the contrary, a series of revitalization policies and long-term development plans in the northeast region have directly improved the quality and accessibility of basic public services, and the relatively low population density also enable centralized and effective service resources allocation. Coupled with higher policy continuity and stability in Northeast China, BPSP has further improved MSI and ultimately transformed into the driving force of PU. Meanwhile, high living costs and competitive pressure in the East, and lower economic development and harsh natural conditions in the West (Xu et al., 2026; Liu et al., 2018), may weaken migrants’ motivation to realize long-term settlement intentions. In other words, even if BPSP increases their willingness to reside, the actual residence behavior of the migrants may still be hindered, and thus the mediating effect of MSI is not obvious. Besides, housing price moderates the mediation most strongly in the East, while hukou restrictions matter most in the West. This may be because housing prices are relatively high and regulation policies are more stringent in East China. In contrast, public service resources in the western region are relatively scarce, and the hukou system determines their priority allocation. This resource allocation gap constrains the positive effect of BPSP, reflecting the slower hukou system reform in the western region. These regional differences underscore that the psychological mediation of MSI is substantially shaped by the broader socio-ecological context. The perceived economic pressures, environmental harshness, and institutional barriers jointly modulate the extent to which public services foster the sense of belonging, security and future expectation that are essential for settlement intentions.

Third, the mediating effect of MSI is significantly positive only in peripheral cities. A possible explanation is that peripheral cities have lower social integration barriers and smaller market fluctuations, making BPSP improvements more effective in enhancing settlement intentions and subsequent urbanization. In contrast, migrants in central cities may face greater social integration obstacles and higher living cost (Lin and Zhu, 2022), dampening their settlement intentions. These findings suggest that improving BPSP in peripheral cities, that is, the equalization of BPSP across cities of different administrative levels, will enhance attractiveness of peripheral cities to migrants, reverse the trend of population clustering towards central cities, and ultimately promote the formation of a scientific and reasonable population urbanization pattern. Moreover, housing price exerts a stronger negative moderating effect on the mediation process in central cities, while the negative moderating effect of hukou restrictions is more pronounced in non-central cities. This is probably because high housing price amplifies economic pressure and uncertainty in central cities, and public service resources are relatively limited and prioritized by the hukou system in peripheral cities. Notably, housing price shows a significant positive moderating effect on the relationship between MSI and PU in peripheral cities. This may be because rising housing price in these cities not only reflects increased living costs but also signals economic development and future growth potential, thereby positively moderating the realization of MSI. These patterns reveal that psychological mediation mechanisms vary by administrative hierarchy. In less congested and peripheral urban environments, the psychological benefits of public services, such as enhanced place attachment and reduced residential uncertainty, are more likely to be converted into durable settlement intentions, whereas in larger central cities, the formation of the same intentions faces greater psychological friction from perceived economic strains and social exclusion. Collectively, these results indicate that the psychological mechanisms of settlement intention are not uniform but are systematically shaped by service types, regional contexts, and urban hierarchies, offering a nuanced psychological map of how policy interventions translate into demographic change.

This study also has some limitations that need further consideration. Firstly, given the multidimensional connotations of basic public services and urbanization, future research could further develop an evaluation system with richer dimensions and more comprehensive indicators, more systematically explore the micro-level psychological processes underlying settlement decisions, and integrate insights from urban psychology, environmental psychology, and public policy to build a more comprehensive understanding of people-centered urbanization. Secondly, given the mobility constraints imposed by the hukou system, a key limitation of this study is that we did not identify “potential voters” who are attracted by the service but give up their migration decision due to hukou barriers This self-selection implies that our estimated coefficients may represent a conservative and underestimated measure of the true public service preferences within the “voting with their feet” framework. Future research needs to consider using tracking data or field experiments to further separate the “blocking effect” of hukou system and the “attraction effect” of public service on migration decisions. Thirdly, this analysis is constrained by the temporal scope of the database, which extends only to 2018. Where conditions permit, future research could further extend the time horizon and employ more recent data to test the temporal robustness of our conclusions. Nevertheless, we wish to emphasize that the primary objective of this study is mechanism identification rather than point-in-time effect estimation. Unlike reduced-form effect sizes, which may vary significantly with changing economic and policy environments, the underlying psychological mechanism through which public service provision shapes settlement intentions and subsequently influences urbanization is theoretically grounded in fundamental human needs and cognitive-affective processes. These psychological pathways represent relatively stable behavioral regularities that are unlikely to be fundamentally altered by the mere passage of time. Therefore, we contend that this theoretical contribution has relatively limited dependence on data timeliness, and it is reasonable to expect that the mechanism holds broadly applicable across different time periods. Finally, we fully acknowledge the limitations of observational studies in providing perfect causal identification. It is worth mentioning that our findings are defined as robust associations consistent with causal explanations, rather than explicit causal estimates. Future research could further employ quasi-experimental designs to strengthen causal inference.

## 6 Policy implications

Based on these conclusions, corresponding policy recommendations are proposed. Firstly, given the positive effect of BPSP on MSI, cities, especially small and medium-sized ones, should prioritize improving BPSP for migrants. Special attention should be paid to eliminate welfare disparities linked to the hukou system and promote public service equalization. This requires strengthening government performance evaluation in public services delivery for migrants and encouraging responsiveness to migrants’ needs. Expanding service coverage to ensure migrants benefit where they live or work is also crucial. Specifically, governments should allocate financial resources based on the permanent population and gradually build a public resource allocation system tailored to the multi-level, diverse, and dynamic needs of migrants. Such efforts can enhance urban inclusiveness and advance new-type urbanization. From a psychological perspective, inclusive public services foster a sense of security and place attachment, which are essential for transforming temporary migrants into permanent urban residents.

Secondly, differentiated policies should be developed to promote the coordinated development of public service and urbanization, and guide rational and orderly population distribution. Heterogeneity analysis advocates for region-specific, city-specific, and multi-tiered policy designs. Specifically, given that the mediating effects of MSI vary across regions, regional differentiated policies should be explored according to local conditions to narrow spatial disparities. Eastern cities should alleviate the suppressive effect of housing prices on settlement willingness, while western cities should focus on improving economic development and natural conditions, and taking the lead in lifting hukou restrictions to unleash service-related dividends. Meanwhile, the central region should transition from quantity-driven to quality-driven development, prioritizing service-quality improvements to support migrants in realizing their settlement intentions, while the northeastern region should maintain its strengths in policy consistency and stability. We further recommend establishing a public service system and a package of supporting policies tailored to city administrative levels and scales. For example, central cities should progressively ease hukou restrictions, regulate housing costs, and ensure public service equalization for low-human-capital employees, rather than focusing solely on high-skilled labor. Peripheral small- and medium-sized cities should prioritize employment-related public services and promote inter-city public service equalization through fiscal transfers and policies, thereby leveraging public service improvements to attract population inflows and contribute to the optimization of regional urbanization patterns. Considering that the mediating effect of MSI is more pronounced for livelihood-related public services, this study further recommend that education, healthcare, social security, and other services directly related to migrants’ life quality should be the focus of local governments in promoting people-oriented urbanization.

Thirdly, we recommend sustained and targeted hukou reform to promote PU. For one thing, we recommend cities developing specific and differentiated household registration system tailored to local conditions. For example, large cities can retain hukou-based regulatory tools to manage population size within a certain range, including the points-based household registration system and differentiated settlement scheme in suburban areas. Small and medium cities can implement flexible and relaxed hukou policies such as “residence equals registration” programs and mutual recognition of residence duration between cities. Moreover, urban agglomerations and metropolitan areas can serve as strategic units for coordinating hukou policies across cities of different sizes, so as to develop differentiated and coordinated management frameworks. For another, it is essential to eliminate the social welfare differences related to hukou system gradually, so as to promote equal access to basic services for rural migrants. Reducing institutional barriers is likely to mitigate the feelings of relative deprivation and social exclusion that currently weaken migrants’ psychological commitment to urban life.

Finally, we recommend adopting city-specific and multi-level approaches to housing price management, along with differentiated housing policies to reduce migrants’ housing costs. For large cities, financial, fiscal, and land-use instruments should be employed to encourage self-occupancy and suppress speculative demand. For small and medium-sized cities, policies should focus on reducing housing inventory and establishing long-term land utilization mechanisms. Governments should also expand the supply of affordable, public rental, and low-rent housing to meet diverse needs. Additionally, flexible housing price regulation policies integrating land supply, credit, and tax measures should guide market expectations and slow rapid housing price increases. By alleviating housing-related psychological stress, such policies can preserve the psychological benefits of public services and reinforce migrants’ intentions to settle.

## 7 Conclusion

This study is the first to investigate the psychological influence mechanism of basic public service provision (BPSP) on population urbanization (PU) through migrants’ settlement intention (MSI). It further examines the moderating effects of housing price and hukou restrictions and its heterogeneity. Specifically, MSI partially and positively mediates the BPSP-PU relationship. Housing price negatively moderates this mediation, while hukou restrictions negatively moderate the BPSP-MSI link but positively moderate the MSI-PU link. Heterogeneity analyses further reveal that these mediating and moderating effects vary by public service types, regions and city administrative levels. This study demonstrates that the pathway from public service provision to urbanization operates through a distinctly psychological channel, migrants’ settlement intention, whose effects are conditioned by economic and institutional factors. These findings hold significant implications for the refinement and application of contemporary policy, suggesting that policy design should pay greater attention to the subjective experiences and psychological integration of the migrants. Understanding the psychological costs associated with institutional exclusion and housing stress can help policymakers design interventions that not only attract migrants but also strengthen their long-term settlement intentions. In conclusion, this study provides a psychologically informed intervention framework for steering urbanization policy towards quality enhancement. It underscores that institutional design should integrate individuals’ psychological expectations, sense of belonging, and perceived life feasibility into governance frameworks, thereby aligning policy incentives with psychological momentum and truly advancing sustainable and human-centered urban development.

## Data Availability

The datasets presented in this article are not readily available because the datasets are part of an ongoing study. Requests to access these datasets should be directed to Zerui Ni, zeruini@hytc.edu.cn; Yan Jin, jinyan@lixin.edu.cn.
